# Rate of Clinical Complete Response for 1 Year or More in Bone-Metastatic Breast Cancer after Comprehensive Treatments including Autologous Formalin-Fixed Tumor Vaccine

**DOI:** 10.1155/2018/4879406

**Published:** 2018-01-22

**Authors:** Fumito Kuranishi, Yuki Imaoka, Yuusuke Sumi, Yoji Uemae, Hiroko Yasuda-Kurihara, Takeshi Ishihara, Tsubasa Miyazaki, Tadao Ohno

**Affiliations:** ^1^Department of Surgery, Innoshima-Ishikai Hospital, Innoshima, Onomichi, Hiroshima 722-2211, Japan; ^2^Cell-Medicine, Inc., 2-1-6 Sengen, Tsukuba, Ibaraki 305-0074, Japan

## Abstract

**Introduction:**

No effective treatment has been developed for bone-metastatic breast cancer. We found 3 cases with clinical complete response (cCR) of the bone metastasis and longer overall survival of the retrospectively examined cohort treated comprehensively including autologous formalin-fixed tumor vaccine (AFTV).

**Patients and Methods:**

AFTV was prepared individually for each patient from their own formalin-fixed and paraffin-embedded breast cancer tissues.

**Results:**

Three patients maintained cCR status of the bone metastasis for 17 months or more. Rate of cCR for 1 year or more appeared to be 15% (3/20) after comprehensive treatments including AFTV. The median overall survival time (60.0 months) and the 3- to 8-year survival rates after diagnosis of bone metastasis were greater than those of historical control cohorts in Japan (1988–2002) and in the nationwide population-based cohort study of Denmark (1999–2007).

**Conclusion:**

Bone-metastatic breast cancer may be curable after comprehensive treatments including AFTV, although larger scale clinical trial is required.

## 1. Introduction

Bone metastases of breast cancer are frequently found in the rib, sternum, vertebrae, or pelvis and are refractory to radiation therapy combined with standardized HER2-binding monoclonal antibody and/or endocrine treatments and, if necessary, cytotoxic chemotherapy comprising a widespread regimen such as cyclophosphamide, epirubicin, and 5-fluorouracil (CEF) [1]. Severe pain from bone metastasis limits the mobility of breast cancer patients and compromises their quality of life. A nationwide population-based cohort study in Denmark (1999–2007) revealed that the 5-year survival rate was only 8.3% for patients with bone metastasis, 2.5% for those with bone metastasis and skeletal-related events (SREs), and 23% for those with bone metastasis but no SREs, whereas the survival was 75.8% for breast cancer patients without bone metastasis [2]. Therefore, bone metastasis strongly influences survival of the breast cancer patients.

However, no effective method has been developed for treatment of bone metastasis. For example, agents such as zoledronate and aromatase inhibitors are effective in limiting further progression of the cancer and can significantly improve overall survival [3] and disease-free survival [4], yet they are not expected to completely cure breast cancer with bone metastasis. To date it is widely believed that bone-metastatic breast cancer is incurable, as Mundy has described: “once tumor cells become housed in the skeleton, cure is no longer possible and only palliative therapy is available” [5].

Autologous formalin-fixed tumor vaccine (AFTV) is the ultimately personalized drug, custom-made after resection of the tumor against the patient's own residual tumor. It has been used to treat patients with chemorefractory tumors since 2002 [6]. The efficacy of AFTV has been reported in a randomized clinical study on hepatocellular carcinoma (HCC) [7], a pilot study and two subsequent phase I/IIa studies on glioblastoma multiforme [8–10], and case reports on advanced glioblastoma [11], malignant fibrous histiocytoma [12], recurrent HCC [13], recurrent peritoneal serous carcinoma [14], uterine cervical small cell carcinoma [15], upper tract urothelial carcinoma [16], and gall bladder cancer and colon cancer [17]. AFTV has been shown to induce cytotoxic T lymphocytes specific to glypican-3, the protein frequently expressed in HCC [18].

In parallel with these small-scale clinical settings, we searched retrospectively patients treated with AFTV in the course of comprehensive treatments for bone-metastatic breast cancer since 2004 in two nearby regional hospitals in Onomichi city, Hiroshima, Japan, in both of which one of the authors (FK) has been working. We encountered an advanced case of breast cancer with bone metastasis in a patient who had shown strong uptake of ^99m^Tc at the location of vertebra Th7 in August 2006. After combined treatments with AFTV, palliative radiation therapy, and adjuvant chemotherapy, the patient's vertebra revealed no uptake of ^99m^Tc in December 2010 [20]; therefore we diagnosed eradication of the bone-metastatic breast cancer. However, our case report attracted the criticism that bone scintigraphy sometimes leads to a false conclusion.

Here we report 20 cases of bone-metastatic breast cancer patients followed in August 2017, including their survival indices which were apparently greater than those of the Denmark study [2] and follow-up confirmation by single-photon emission computed tomography combined with X-ray computed tomography (SPECT-CT) or by positron-emission tomography combined with CT (PET-CT) of the clinical complete response (cCR) of three breast cancer cases showing long-term cCR of metastatic bone cancer after comprehensive treatments including AFTV. To our knowledge, the rate of cCR maintained for 1 year or more has not been documented in breast cancer patients with bone metastasis.

## 2. Patients and Methods

Breast cancers are normally treated according to established guidelines [1]. After resection of the primary cancerous tissue and according to the expression level of HER2, estrogen receptor (ER), and progesterone receptor (PgR) in the carcinoma cells, we treated patients with anti-HER2 antibody, endocrine treatments or aromatase inhibitors, combined with cytotoxic agents, and if applicable X-ray irradiation. The dose of radiation was selected case-by-case, with 50–60 Gy administered to the main target lesion and/or 30–36 Gy to any bone-metastatic lesions to suppress bone-derived pain and to avoid induction of further skeletal-related adverse events. Regular screening of bone metastasis of breast cancer depends on ^99m^Tc bone scintigraphy. The precise locations of advanced bone metastases were determined by single-photon emission computed tomography combined with X-ray computed tomography (SPECT-CT) or by positron-emission tomography combined with CT (PET-CT). If patients required any extra-standard treatments, frequently required in bone-metastatic breast cancer cases or in the cases with high probability of bone metastasis that was estimated by the skilled medical doctor (FK), we introduced AFTV treatment.

The AFTV was prepared individually for each patient from their own formalin-fixed and paraffin-embedded breast cancer tissues as has been described for cases of glioblastoma multiforme [8]. The prepared AFTV was injected intradermally during the course of comprehensive treatment, whenever possible before the start of fractionated bone-irradiation. Each course of AFTV treatment consists of three intradermal injections every 2 weeks. To prepare AFTV for one course, we need 2.0 g of paraffin-removed breast cancer tissue specimen. If the amount of resected primary tumor was sufficient to prepare AFTV for multiple courses, two or more courses of treatment with AFTV were administered. Adverse effects of treatment with AFTV were graded according to the National Cancer Institute Common Toxicity Criteria version 2 [21] or, later, according to the Common Terminology Criteria for Adverse Events (CTCAE) v3.0.

The present treatment with AFTV has been approved by the ethics authorities of JA Onomichi General Hospital and Innoshima-Ishikai Hospital, both of which are located in Onomichi city, Hiroshima, Japan. Informed consent was provided by all of the patients for the treatment with AFTV and publication of the clinical data. The present retrospective study was registered to the UMIN clinical trials registry, Japan, as UMIN000029726.

## 3. Results

Among 119 breast cancer patients so far given comprehensive treatment including AFTV between 2004 and 2013, 20 bone-metastatic cases were screened, 16 of which bore bone metastasis before AFTV treatment and four revealed bone metastasis after AFTV treatment (Cases #10, #15, #17, and #18) (Table 1). The eradication of a 3 cm diameter bone metastasis in vertebra Th7 of Case #6 was previously reported based on the simple observation of whole body ^99m^Tc bone scintigraphy [20]. We confirmed maintenance of her cCR status by SPECT-CT after more than 6 years as described in the Case Presentations. Two new cases (Cases #17 and #20) are shown in Table 1 who maintained long cCR status. They remain well with no evidence of recurrence or new metastasis, the former for 18 months and the latter for 17 months. These 3 cases, all showing solitary bone metastasis, are described more fully in the following section.

In Case #18, complete local control of the metastatic vertebra L5 was successful for 22 months. However we observed parallel multiple metastases in lymph nodes. Case #19 achieved cCR after AFTV therapy and radiation therapy (36 Gy) and maintained their cCR status for 11 months (bone metastases in both of the vertebrae L3 and L5 disappeared on PET-CT), but then new lymph node metastasis was found, though after irradiation to the new lesion the patient remains well in February 2017 without any signs of lumbago and recurrence. We also found six cases with long-term stable disease (SD) in the metastatic bone(s) but without any new lesions. Local SD status was maintained for variable times, from the shortest one at 5 months (Case #1) to the longest one at more than 80 months (Case #16). Notably, Cases #4, #11, #13, and #16 bore multiple bone metastases before treatment with AFTV but have since maintained SD for longer than a year. The other nine cases were classified as progressive disease (PD), because AFTV showed no effect on growth of their bone metastases.

If “1-year cCR,” defined as whole body cCR maintained for 12 months or more, is acceptable, the rate of 1-year cCR reached 15% (3/20) among breast cancer patients with bone metastasis given comprehensive treatment including AFTV. In addition, adding to 1-year cCR cases, those with SD of 1-year duration in the metastatic bones, the control rate of bone metastasis reached 45% (9/20).

**Figure 1:**
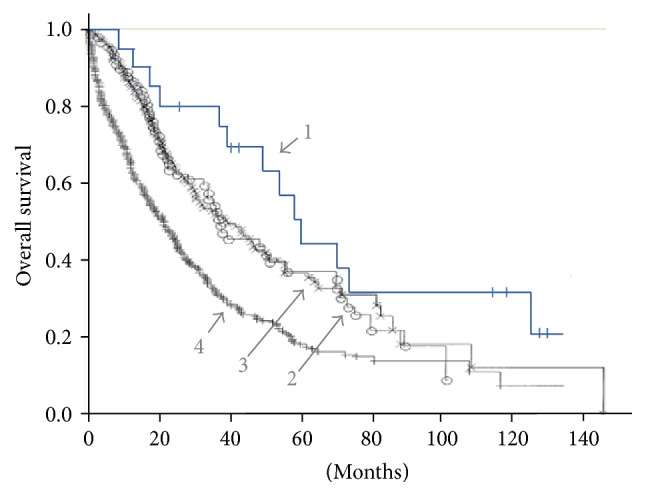
Overall survival (OS) of the present cohort treated comprehensively including AFTV and the historical control cohorts in Japan. Line 1, the present cohort (*n* = 20) with median OS, 60.0 months. Lines 2, 3, and 4, Japanese historical controls taken from Koizumi et al., Figure 2 in [19]. Line 2, patients with solitary sternal metastasis (*n* = 98), median OS, 39.5 months. Line 3, patients with solitary metastatic bone lesion other than sternum (*n* = 191), median OS, 41.4 months. Line 4, patients with multiple metastatic bone lesions (*n* = 414), median OS, 22.6 months.

As shown in Figure 1, the overall survival (OS) after first diagnosis of bone metastasis was calculated and plotted on a Kaplan-Meier curve for all patients listed in Table 1. Median OS and 5-year survival rate were 60.0 months and 50.0% (95% CI: 48.8–71.3 months), respectively. We compared the Kaplan-Meier curve with those of historical controls (gray lines in Figure 1) reported by Koizumi et al. [19] whose patients have been treated comprehensively but have never been treated with AFTV since AFTV was not available for breast cancer patients before 2003 in Japan. Our curve (line 1) looks more favorable than line 2 (Koizumi et al.'s patients with solitary sternal metastasis, *n* = 98), line 3 (solitary metastatic bone lesion other than sternum, *n* = 191), and of course line 4 (multiple metastatic bone lesions, *n* = 414), all taken from Figure 2 of [19]. Since there was no difference in survival between line 2 and line 3, we combined the number of patients at risk each year for these two Koizumi et al.'s cohorts available in the Table 5 of [19] and then compared with the patients at risk in the present cohort. Differences of population ratio of survival at each year were statistically significant between the present and Koizumi et al.'s combined cohort, at least from 3 to 8 years of survival (Table 2).

**Figure 2:**
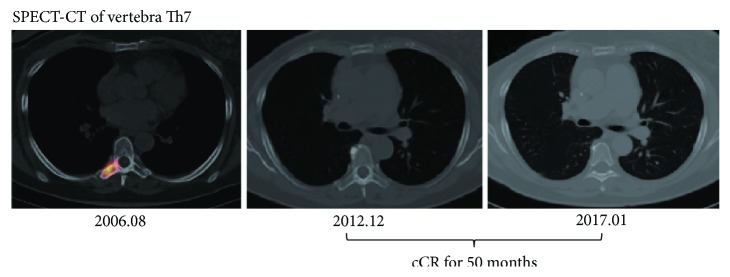
Follow-up SPECT-CT of vertebra Th7 of Case #6 (assigned as cCR Case 1) [20]. No recurrence in vertebra Th7 was observed.

Also, the data compare favorably with those described from a nationwide population-based cohort study in Denmark [2]. In patients after diagnosis of bone metastasis but no SREs during the follow-up in Denmark, median OS and 5-year survival rate were 17 months and 23%, respectively (read-out data from Figure 2 of [2], *n* = 772 for bone-metastatic breast cancer patients without SREs). Both of these concrete data are located outside of the lower 95% CI line of the Kaplan-Meier curve of our present cohort. There is a difference between, for example, the two sets of 5-year survival data (the present 50% versus the 23% reported in the Figure 2 of the Denmark study, *p* = 0.0052 by Chi square test).

None of the patients in Table 1 experienced any severe complication closely related to AFTV treatment. The adverse events observed consisted of local erythema, induration, and swelling at the injection sites. These effects corresponded to Grade 1 toxicity in all cases. The low grade adverse events directly associated with the AFTV treatment were quite similar to those reported in cases of glioblastoma multiforme treated with AFTV [9, 10].

## 4. Case Presentations

### 4.1. Case #6

Case #6, a 52-year-old woman, was first treated with one course of AFTV and concomitant palliative radiation therapy (36 Gy) in August–December 2006, followed by adjuvant chemotherapy comprising six courses of CEF, zoledronate, and aromatase inhibitors (anastrozole and exemestane) [20]. To our surprise, however, the original breast carcinoma was classified as “triple negative” by the primary surgeon. Apparently the aromatase inhibitors were misprescribed, something that also happened in Case # 10 in Table 1.

To confirm whether or not there was any recurrence, Case #6 was precisely examined, not by regular ^99m^Tc bone scintigraphy, by SPECT-CT in December 2012 and again in January 2017. No sign of recurrence or new metastasis in the patient's bone (Figure 2) or blood tumor markers was reported, showing that her cCR status was maintained for 50 months. We therefore assigned this case as tomographically confirmed cCR Case 1.

### 4.2. Case #17

**Figure 3:**
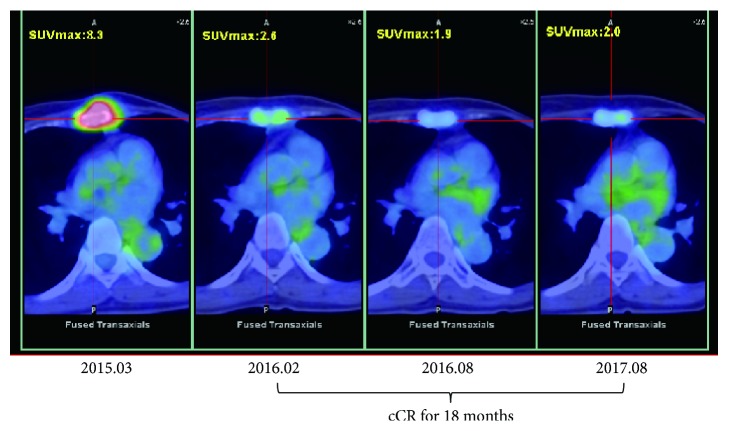
PET-CT of the sternum of Case #17 (assigned as cCR Case 2). The cCR status has been maintained for 18 months to date.

**Figure 4:**
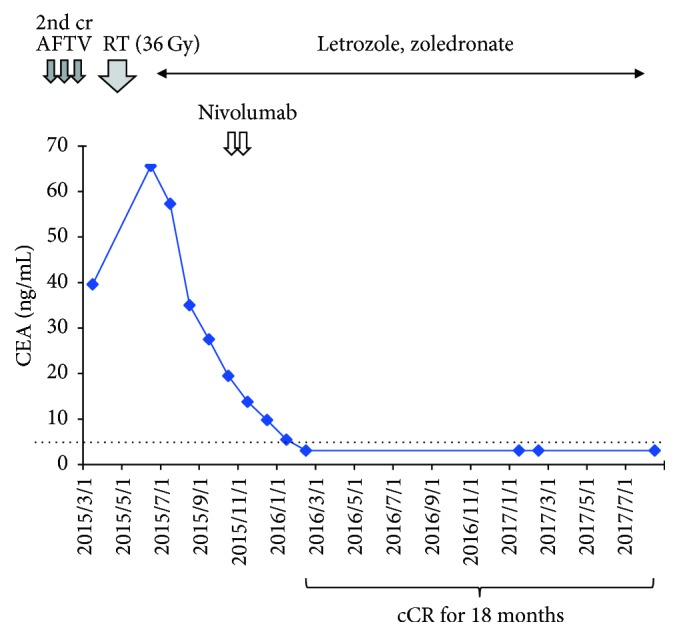
Alteration of blood CEA level of Case #17 after comprehensive treatments including AFTV. 2nd cr AFTV, 2nd course of AFTV treatment; RT, radiation therapy. Dotted line indicates basal level of blood CEA in normal subjects.

A 50-year-old woman was found to have a 2.5 cm mass of tubular carcinoma in her left breast by magnetic resonance imaging (MRI). The carcinoma was resected in March 2009. No accompanying lymph node metastasis was observed. We found the tumor was HER2(−), ER(+), and PgR(−). Four months after resection, the patient agreed with one course of AFTV as the first-line adjuvant therapy. The response in delayed-type hypersensitivity test to her own carcinoma became strongly positive (40 × 35 mm erythema with 5 × 5 mm induration). We then treated her with the aromatase inhibitor, anastrozole. Six years later, in March 2015, PET-CT revealed bone metastasis to the sternum (Figure 3, left) which was confirmed by aspiration biopsy cytology as class V metastatic ductal carcinoma. We treated her again with a second course of AFTV and palliative radiation therapy (36 Gy/12 fractions/19 days) together with treatment with another aromatase inhibitor, letrozole (2.5 mg/day) in combination with zoledronate (4 mg/month), and an additional 2 shots of nivolumab (a vial, 40 mg, per 48 kg body weight) in October and November 2015. She was diagnosed as cCR by PET-CT in February 2016. We confirmed her cCR status in August 2016 and again in August 2017 (Figure 3). Alteration of her blood CEA level (Figure 4) suggests that the combined treatments probably eradicated the metastatic breast carcinoma. Up to date she has maintained her cCR status for 18 months; thus we assigned this case as cCR Case 2.

### 4.3. Case #20

**Figure 5:**
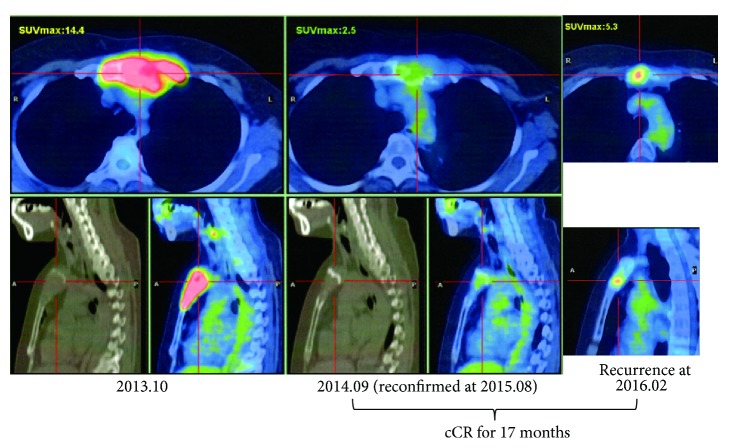
PET-CT of the sternum of Case #20 (assigned as cCR Case 3). A huge bone metastasis of breast carcinoma was observed. The length of cCR status was calculated between the reconfirmation of disease-free status on September 3, 2014, and the diagnosis of local recurrence on February 9, 2016.

A 46-year-old woman with stage II breast cancer (scirrhous ductal carcinoma) was operated on in July 2000. She had been treated with toremifene citrate, 40 mg/day. Left axillar recurrence was found 13 years later by ultrasonography and biopsy. PET-CT revealed a huge bone metastasis in the sternum (Figure 5, left). She was treated with radiation (60 Gy) and concurrently with three AFTV injections (one course) between October and December 2013, one additional AFTV injection (1/3 course) in September 2015, and then zoledronate (4 mg/month) and tamoxifen (20 mg/day) or letrozole (2.5 mg/day). Follow-up PET-CT (September 2014) showed no sign of residual carcinoma (Figure 5, middle); therefore we consider that the patient entered into cCR status which was reconfirmed in August 2015. To avoid possible recurrence, she received nivolumab administration (a vial, 40 mg, per 51 kg body weight) three times at 3-week intervals during October-November 2015. However, a small hot spot was observed in the sternum in a PET-CT image taken in February 2016 (Figure 5, right) and local recurrence in the sternum was confirmed by MRI in March 2016, suggesting that cCR status had been maintained for 17 months by the time of local recurrence in the sternum. Therefore we assigned this case as cCR Case 3, since she maintained the cCR status for more than a year. Additional pin-point irradiation (60 Gy) to the sternum soon suppressed the recurrence and she remains well at present (confirmed by February 2017).

## 5. Discussion

Before encountering Case #6, assigned as cCR Case 1 (Figure 2), we previously observed approximately 300 cases of mammary carcinoma with bone metastasis over a period of 10 years up to the end of 2006. All these patients showed a downhill course resulting in fatality despite administration of intensive chemoendocrine-radiation therapy. Eradication of bone-metastatic breast carcinoma had never been successfully achieved in any of our patients, although we have observed in a separate retrospective study that AFTV treatment, added after 2004 on the standardized treatments for breast cancer patients without bone metastasis, increased significantly the number of white blood cells and lymphocytes, CD3^+^ T cells, percentage of Th1 in CD4^+^ T cells, and ratio of Th1 and regulatory T cells (Supplementary Table (available here)). As is well-known, X-ray irradiation of bone metastasis is a palliative treatment, a conclusion drawn from the results of 16 randomized trials, 20 prospective studies, 5 retrospective studies, and 22 other articles, involving a total of 8,051 patients [22]. Therefore, it is extremely rare to observe eradication of skeletal metastasis of breast cancer which is refractory to standardized treatment. Particularly in the trunk area, conventional full dose irradiation (60 Gy), which may cause late radiation injury to major organs, has been avoided, and lower doses such as 36 Gy used for pain reduction are unable to eradicate the skeletal metastasis. Moreover, no adjuvant therapeutic regimen has been found which can effectively treat bone metastasis of breast cancer.

However, following the introduction of AFTV in the comprehensive treatment of advanced breast cancer, we have become aware that at least some of the breast cancer patients with bone metastasis may escape the fateful downhill course as shown in Table 1. Although transient shrinkage of the bone-metastatic lesion could be observed by ^99m^Tc bone scintigraphy, we classified partial-response (PR, assumed) into stable disease (SD) in the column of Table 1, “best response of bone-meta after AFTV treatment,” because of unreliable quantification of the size of the bone metastasis by ^99m^Tc bone scintigraphy. For example, in Case #1, the metastasis in vertebra Th4 was almost diminished when analyzed by bone scintigraphy after treatment with three courses of AFTV, docetaxel, and aromatase inhibitors (possibly entered into PR status). The patient maintained this status for 5 months and then developed two new metastases in right ribs 3 and 4. Case #2 with multiple bone metastases of triple-negative papillotubular carcinoma was unable to undergo treatment with any cytotoxic agents because of renal failure. We resected the rib with metastatic lesion. She developed recurrence in the remaining ribs and new metastases in the cervical spine, but short-term transient CR of the recurrent bone metastases was revealed by bone scintigraphy after two courses of AFTV treatment and irradiation (36 Gy). After these complicated experiences, we encountered Case #6 which we reported previously based on the results of ^99m^Tc bone scintigraphy [20]. This time we were able to confirm continuation of her cCR status on her follow-up SPECT-CT up to 50 months (Figure 2).

All three of the cCR cases had carried solitary bone metastases, two cases (Case #6 and #20) before treatment with AFTV and one case (Case #17) after treatment with AFTV (Table 1). Although it has been reported that “a solitary bone metastasis can often be successfully treated, and long-lasting complete remission is not unusual” in a review of oligometastatic breast cancer [23], no numerical percentage values appeared in the original report [19] cited in the review. In the literature up to 2013, we recently found one case report describing complete response in breast cancer metastatic to liver and bone [24]. Together with our previous experience up to the end of 2006 on approximately 300 bone-metastatic breast cancer cases, this rare case report implies that the percentage of patients who experience complete response of bone-metastatic breast cancer is likely to be less than 1% in our bone-metastatic historical cohort.

Similar to Case #6, we observed that in Case #18, complete local control of the initial bone metastasis was achieved and lasted for 13 months but was accompanied in parallel with metastases to lymph nodes and other bones. Case #19 maintained complete remission of the bone metastasis for 11 months, but a new lymph node metastasis appeared after this period (Table 1). We did not count, of course, Case #18 or Case #19, as cCR cases, since medical doctors in the regional hospitals did not positively evaluate complete local control of the initial bone metastases but rather pointed out the new metastases appearing in other organs within a year, saying that a cCR term of less than a year is too short to convince their patients that the treatment is effective. Therefore, we defined cCR as “1-year cCR” as described in Results. In the treatment with AFTV, we did not adopt the common positive criterion, pathological complete response, since it is usually impossible to obtain a biopsy sample from metastatic bone.

The Kaplan-Meier curve from the data of OS after the first diagnosis of bone metastasis (Figure 1) was apparently different from that described in the nationwide population-based cohort study in Denmark (1999–2007) in which 35,912 newly diagnosed breast cancer patients were identified from January 1, 1999, to December 31, 2007, in the Danish National Patient Registry. Of these, 1,494 developed bone metastases and of these a further 722 developed both bone metastases and SREs and 772 bore bone metastases without SREs [2]. Therefore, we consider the Kaplan-Meier curve of OS and 5-year survival rate from diagnosis of bone metastasis without SREs (Figure 2 in [2]) is concrete data derived from the huge cohort.

Another set of concrete data was reported from 703 patients with bone metastasis in which the median survival time from the onset of skeletal metastasis was 3.3–3.6 years for patients with solitary sternal metastasis or solitary metastatic bone lesions other than sternum and 2 years for patients bearing multiple metastatic bone lesions. These data are presented in Figure 2 of the paper by Koizumi et al. [19], as cited in Figure 1. The present cohort shown in Table 1 and in Figure 1 revealed longer median OS and higher 5-year survival rate when compared to these two concrete datasets in [2, 19], although results from the present cohort do not necessarily prove the efficacy of AFTV since the present cohort is small and therefore probably includes unnoticed patient selection bias. For example, all the patients in Table 1 have undergone resection of breast cancer at an appropriate time, but in Cases #1 and #6 they were surgically operated on after the first diagnosis of bone metastasis, even though resection of metastatic breast cancer is not recommended for advanced stage IV patients. These records therefore imply that ad hoc selection has occurred at the time of obtaining informed consent from the breast cancer patients with bone metastasis. A larger scale clinical study with appropriate control patients will be desirable to confirm the efficacy of AFTV treatment on bone metastases of breast cancer. Nevertheless, our data suggest that AFTV therapy should have contributed at least partly through the three cCR cases to the long median OS, 60 months from the diagnosis of bone metastasis, of the present cohort. Statistically significant differences of population ratio of survival at each year between the present cohort and Koizumi et al.'s combined cohort in Japan (Table 2) are fairly meaningful for the estimation that treatment of bone-metastatic breast cancer with additional immunotherapy using AFTV is considered to be well justified.

With regard to examining the reduction curve of blood CEA level (Figure 4), the effect of the 2 additional shots of nivolumab, the potent immune check-point inhibitor, was unclear. Also the effect of additional treatment with nivolumab for Case #20 apparently did not suppress the recurrence in the sternum (Figure 5). From these two poor experiences, it is too early to estimate the efficacy of the immune check-point inhibitor on bone metastasis of breast cancer. Much more experience must be accumulated of the use of the immune check-point inhibitor to evaluate its effect on breast cancer, as in melanoma and lung carcinoma [25].

## 6. Conclusion

The rate of 1-year cCR of 15% suggests that bone-metastatic breast cancer may be partly curable after comprehensive treatments including AFTV. The probable contribution of AFTV to OS should be taken into consideration when planning comprehensive therapeutic courses for the treatment of advanced breast cancer patients with bone metastasis, although a larger scale clinical study is required.

## Figures and Tables

**Table 1:** Characteristics of the present cohort (cut-off, August 31, 2017).

Case #	Age, at diagnosis	Primary resection	First diagnosis of bone metastasis	Receptor status	Any metastasis before AFTV treatment	Treatments after primary resection and before diagnosis of PD	Best response of bone metastasis after AFTV treatment	Outcome by Feb & Aug 2017	OS after the first diagnosis of bone metastasis (months)
1	59	2004/4/21	2004/2/9	HER2(−), ER(++), PgR(++)	Multiple bones (more than 30), liver	Chemo, AFTV 4 cr, chemo, RT (30 Gy), aromatase inhib	SD for 5 months	Dead 2008/02/21	49

2	74	2001/2/2	2003/1/15	HER2(−), ER(−), PgR(−)	Rib, neck, sacrum	Rib resec, RT (30 Gy), chemo, AFTV 2 cr	PD	Dead 2009/01/15	73

3	50	1999/5/10	2005/7/11	Not tested	Brain, pelvis, sacrum	Brain operation, RT to brain (35 Gy), AFTV 1 cr, aromatase inhib, RT to sacrum (30 Gy)	SD for 12 months	Dead 2008/05/07	34

4	56	1999/6/18	2003/3/6	HER2(+), ER(+), PgR(−)	Lymph node (19/20), sternum, lung (multiple), liver, brain (multiple), pelvis	RT (50 Gy), resec, aromatase inhib, chemo, resect, RT (35 Gy to pelvis, 35 Gy to brain), AFTV 1 cr + chemo	SD for 13 months	Dead 2007/07/28	54

5	52	2003/7/8	2004/11/10	HER2(−), ER(−), PgR(−)	Lymph node, bone (multiple), lung	RT (30 + 30 Gy), chemo, AFTV 4 cr + RT (Th6 30 Gy, Th11 30 Gy)	PD	Dead 2009/8/30	58

6	52	2006/8/7	2006/7/31	HER2(−), ER(−), PgR(−)	Vertebra Th7	AFTV 1 cr, RT (36 Gy), chemo, aromatase inhib (misprescription), zoledronate	CR for 50 months or more	Ongoing	128+

7	42	2004/3/5	2006/6/24	HER2(−), ER(+), PgR(+)	Liver, lung, chest wall, lymph nodes, pelvis	RT (30 Gy), chemo, resec, chemo + aromatase inhib, RT (35 Gy), zoledronate, AFTV 3 cr, chemo	PD	Dead 2007/11/18	17

8	45	2001/3/9	2007/3/1	HER2(−), ER(−), PgR(++)	Bone (multiple)	Resec, aromatase inhib, zoledronate, AFTV 3 cr, RT (30 Gy)	PD	Dead 2012/11/16	70

9	71	2004/3/23	2006/12/15	HER2(−), ER(+), PgR(−)	Bone (multiple), skin, lymph node, chest wall	Chemo, aromatase inhib, AFTV 1 cr, chemo	PD	Dead 2008/7/28	20

10	62	2007/6/16	2010/1/28	HER2(−), ER(−), PgR(−)	Lymph node (2/7) [bone (multiple) after AFTV]	Chemo, AFTV 1 cr, chemo, aromatase inhib (misprescription), zoledronate	PD	Dead 2010/9/20	8

11	46	2004/3/10	2007/5/14	HER2(−), ER(++), PgR(++)	Lymph node, lung, rib (2 sites), pedicle of thoracic vertebra	AFTV 4 cr, zoledronate, aromatase inhib, chemo	SD for 55 months	Ongoing, bearing bone-meta	118+

12	75	1992/10/7	2007/5/25	HER2(−), ER(−), PgR(−)	Rib	Chemo, resec, AFTV 1 cr	PD	Dead 2008/5/23	12

13	64	1998/5/12	2006/6/14	HER2(+++), ER(+++), PgR(+++)	Chest wall, bone (multiple)	Resec, chemo, aromatase inhib, RT (28 Gy, 30 Gy, 30 Gy), zoledronate	SD for 25 months	Ongoing	130+

14	61	1999/6/28	2004/12/16	HER2(+++), ER(+), PgR(−)	Lung, vertebra Th10	Resec, AFTV 1 cr, TAE, chemo, zoledronate	PD	Dead 2015/4/4	125

15	39	2007/11/9	2008/12/9	HER2(−), ER(−), PgR(+++)	Lymph node (23/24), pelvis	Chemo, AFTV 1 cr, zoledronate, RT (30 Gy)	PD	Dead 2013/11/12	60

16	52	2000/6/30	2007/9/27	HER2(+), ER(−), PgR(++)	Lymph node (1/26), bone (multiple)	RT (60 Gy), AFTV 1 cr, zoledronate	SD for more than 80 months	Ongoing	114+

17	50	2009/3/18	2015/6/18	HER2(−), ER(+), PgR(−)	Sternum	AFTV 2 cr, aromatase inhib, RT (36 Gy), zoledronate, anti-PD-1 Ab	CR for 18 months or more	Ongoing	26+

18	56	2011/3/11	2013/8/9	HER2(++), ER(+++), PgR(+++)	Vertebra L5, lymph node (1/5)	AFTV 2 cr, RT (30 Gy to vertebra + 50 Gy to axilla), chemo, zoledronate, tumorectomy of chest wall meta, aromatase inhib, RT (40 Gy) to lymph node	Bone local control for 22 months, but clinically PD	Dead 2016/10/17	39

19	64	2004/12/2	2013/9/4	HER2(+++), ER(−), PgR(−)	Lymph node (4/5), vertebrae L3, L5	Chemo, G-CSF, RT (60 Gy to neck lymph node), trastuzumab, AFTV 1 cr, RT (36 Gy) to vertebra L3, vertebra L5, zoledronate, resect (chest wall), RT (60 Gy) to chest wall, RT (60 Gy) to lymph node	CR for 11 months, then PD	Ongoing	42+

20	46	2000/7/14	2013/10/20	HER2(−), ER(+), PgR(++)	Sternum, lymph node	Toremifene, RT (60 Gy), AFTV 2 cr, zoledronate, anti-PD-1 Ab	CR for 17 months, then PD	Ongoing	40+

AFTV, autologous formalin-fixed tumor vaccine; chemo, chemotherapy; CR, complete response; cr, course; inhib, inhibitor; meta, metastasis; OS, overall survival; PD, progressive disease; resec, resection; RT, radiation therapy; SD, stable disease.

**Table 2:** Comparison between the present cohort and the historical control, Koizumi et al.'s combined cohort, in Japan.

Survival (years)	0	1	2	3	4	5	6	7	8	9	10
Present cohort											
Number at risk	20	18	16	14	11	8	6	5	5	5	3
Koizumi et al.'s combined cohort											
Number at risk (solitary, sternum + other, from Table 5 of [19])	289	222	157	106	75	53	38	19	8	4	2
Difference of population ratio at each year											
Chi square test, *p* =				0.003	0.005	0.019	0.037	0.003	<0.0001		

## Abbreviations

AFTV: Autologous formalin-fixed tumor vaccine
cCR: Clinical complete response
CEF: Cyclophosphamide, epirubicin, and 5-fluorouracil
CT: X-ray computed tomography
HCC: Hepatocellular carcinoma
MRI: Magnetic resonance imaging
PET-CT: Positron-emission tomography combined with CT
SPECT-CT: Single-photon emission computed tomography combined with CT
SREs: Skeletal-related events.

## Supplementary Materials

Supplementary Materials Supplementary Table: peripheral blood cell counts in breast cancer patients before and after the first course of AFTV vaccination.
