# Supplementary material for: Rate of Clinical Complete Response for 1 Year or More in Bone-Metastatic Breast Cancer after Comprehensive Treatments including Autologous Formalin-Fixed Tumor Vaccine
Source: Int J Breast Cancer. 2018 Jan 22;2018:4879406. doi: 10.1155/2018/4879406 (PMC5822812; doi:10.1155/2018/4879406)
Supplement: Supplementary Materials — Supplementary Table: peripheral blood cell counts in breast cancer patients before and after the first course of AFTV vaccination. [file 4879406.f1.pdf]

SUPPLEMENTARY TABLE: Peripheral blood cell counts in breast cancer patients before and after the first course of AFTV vaccination.

Breast cancer patients (without bone metastasis) have been submitted for treatment with AFTV. After surgery, chemotherapy, and sufficient time to recover their immunological function, we quantified T-cell populations in the peripheral blood of 39 patients before and after the AFTV treatment. Among them, 32 patients exhibited a change in the delayed-type hypersensitivity response from negative to positive to the fixed carcinoma tissue fragments which were not including any immune adjuvant. We examined change of peripheral blood T-cell population in the 32 patients just before the first injection of AFTV and 2 weeks after the third injection of AFTV (the first course of AFTV treatment). The blood samples were collected individually from each patient. Regular flow cytometric analyses were performed for the T cell subtype counting. Regulatory T cells (Treg) were determined as % of $CD4^+CD25^{++}FoxP3^+$ cells in $CD4^+$ T cells.

Cell counts and ratios	(1) Before			(2) After			(3) After/Before ratio			For mean of (3) 99% CI (*) or 95% CI		p <
	mean		SD	mean		SD	mean		SD	lower limit	upper limit	
WBC/μL	5559	±	1503	5977	±	1309	**1.10**	±	0.18	1.02*	1.19*	0.01
Ly/μL	1873	±	565	2004	±	573	**1.10**	±	0.22	1.01	1.18	0.05
$CD3^+$ T cells/μL	1224	±	426	1303	±	422	**1.10**	±	0.21	1.02	1.17	0.05
$CD4^+$ T cells/μL	446	±	200	480	±	196	1.09	±	0.25	0.997	1.18	ns**
$CD8^+$ T cells/μL	434	±	207	447	±	217	1.08	±	0.29	0.98	1.19	ns
Th1 (%) in CD4+ T cells	31.2	±	10.5	32.4	±	10.1	**1.05**	±	0.13	1.004	1.1	0.05
Th1/Th2	11.5	±	8.1	10.4	±	6.4	1.01	±	0.41	0.87	1.16	ns
Treg (%) in CD4+ T cells	7.21	±	1.84	6.8	±	1.93	0.95	±	0.18	0.89	1.02	ns
Th1/Treg	4.7	±	2.38	5.38	±	3.29	**1.14**	±	0.27	1.01*	1.27*	0.01
CD8+ T cells/Treg	15.2	±	6.8	16.4	±	10.4	1.06	±	0.27	0.96	1.15	ns

Bold figures indicate statistically significant difference from 1.00.

* CI, confidence interval. ** ns, not significant.
